# First evaluation of in-patient dose calculation accuracy on a C-arm Linear Accelerator with advanced Cone-Beam computed tomography (CBCT) imaging

**DOI:** 10.1016/j.phro.2026.100991

**Published:** 2026-05-13

**Authors:** Kenneth W. Gregg, Theodore Arsenault, Sagar Regmi, Kyle O’Carroll, Beatriz Guevara, Meiying Xing, Runyon Woods, Atefeh Rezaei, Rojano Kashani, Lauren Henke, Alex Price

**Affiliations:** a Department of Radiation Oncology, University Hospitals Seidman Cancer Center, Cleveland, OH, USA; b Department of Biomedical Engineering, Case Western Reserve University, Cleveland, OH, USA; c Case Western Reserve University School of Medicine, Cleveland, OH, USA

**Keywords:** Direct CBCT Dose Calculation, TrueBeam, HyperSight

## Abstract

**Background and Purpose:**

Innovations in on-board Cone-Beam Computed Tomography (CBCT) show promise for accurate dose calculation due to higher CT number precision compared to prior C-arm CBCT systems. We prospectively investigated dose calculation accuracy in patients imaged using a C-arm linac equipped with an advanced CBCT imaging solution utilizing iterative reconstruction algorithms.

**Materials and Methods:**

For image acquisition, 37 patients underwent both a CT-simulation (CT-sim) and high-quality C-arm CBCT in their treatment position. A total of 39 plans (2 patients multi-site) across four body sites (10 Head/Neck, 9 Thorax, 10 Abdomen, 10 Pelvis) were developed on the clinical CT-sim image and recalculated on the CBCT. Dose distributions were compared using 3D gamma analysis passing rates (GPR) using 1%/1mm local criteria and 10% or 50% low-dose thresholding (10%TH, 50%TH). Percent difference in target dose-volume histogram (DVH) metrics D99%, D_mean_, D1%, V100%, and V95% were calculated for comparison.

**Results:**

The CBCT to CT-sim dose calculation comparisons overall GPR with 10%TH averaged 96.2 ± 3.6% and 50%TH GPR average was 95.0 ± 6.9%. Overall, 36 plans at 10%TH and 32 plans at 50%TH achieved > 90% GPR. Percent difference in target DVH metrics averaged 0.15 ± 1.24%, 0.52 ± 1.01%, 0.86 ± 1.29%, −0.92 ± 4.84%, and −0.31 ± 2.42% for D99%, D_mean_, D1%, V100%, and V95%, respectively. Only 9 of 195 (4.6%) target DVH metrics changed by > 5%.

**Conclusion:**

C-arm CBCT dose calculation was comparable to standard-of-care CT-sim in most patients, demonstrating the ability to be used for clinical evaluation and decisions. Direct dose calculations in settings of minimal anatomical motion were most robust**.**

## Highlights

• High-quality cone-beam computed tomography yields accurate dose calculation.
• 77/78 evaluated organ-at-risk dose statistics changed < 5%
• 186/195 evaluated target dose statistics changed < 5%
• All cases gamma pass rate > 90% with 2%/1mm criteria at 10% threshold.

## Graphical abstract

**Figure:**
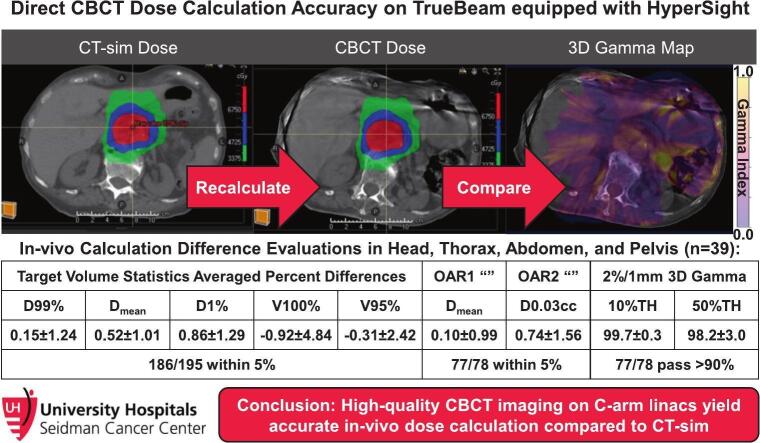

## 1 Introduction

Using CT number (Hounsfield Unit, HU) to electron density mapping in CT simulation (CT-sim) is the clinical standard for inhomogeneity correction in modern external beam dose calculation engines.[1] On-board cone-beam computed tomography (CBCT) has primarily been used for patient alignment in precision treatments for the past few decades. Early CBCT units had smaller field-of-view (FOV), limited image quality, and relative lack of CT Number accuracy compared to CT-sim, making these images unsuitable for precise dose calculation or planning.[2], [3] To overcome these barriers for on-treatment plan adjustments, techniques like CBCT-guided adaptive radiotherapy (ART) initially utilized deformable image registration between CBCT and underlying CT-sim to assign CT number for inhomogeneity correction, creating a synthetic CT (sCT).[4] However, dose errors up to 15.9% have been reported using these methods in lung phantom studies with variable target size, highlighting limitations of this methodology.[5].

Recently, advances in CBCT have enabled direct CBCT dose calculation in ART workflows for an O-ring treatment platform by introducing larger panel geometry (86x43cm^2^ over 43x43cm^2^), high-speed acquisition (6-second over 16.6-second), and iterative reconstruction techniques with scatter correction.[6] These imaging improvements resulted in higher quality images and acceptable CT Number fidelity across disease sites, including gamma passing rates (GPR) > 90% at the 1%/1mm level,[6], [7], [8], [9], [10] or GPR < 85% in cases not suitable for dose calculation due to tissue changes.[11] This led to FDA-clearance of the imaging unit on ring-gantry systems for primary plan calculation in adults in 2023.[12] However, a representative sample survey reported that less than 25% of clinics in the United Kingdom have ring-gantry designs available, limiting the global impact of this advancement.[13].

Recently, similar technological advances have become available on C-arm CBCT systems, with higher acquisition speed (1.5 rpm over 1.0 rpm), greater active imaging area (43x43cm^2^ over 43x30cm^2^), and new scatter correction techniques with iterative reconstruction algorithms. Notably, these improvements on the C-arm platform are limited to slower acquisition speeds and smaller panel geometry compared to the ring-gantry system. Image quality characterization benchmarks on the C-arm CBCT system demonstrated improved high-contrast resolution, low-contrast visibility, and uniformity.[14] CBCT imaging using iterative reconstruction has shown to increase HU precision in-phantom on the C-arm linac over conventional Feldkamp-Davis-Kress (FDK) reconstructions, and dose calculations showed maximum planning target D98 deviation of 3.3% compared to gold-standard CT-sim.[15] However, in-phantom image quality and dose calculation studies lack patient geometry and motion exhibited in daily patient imaging.

Here, we prospectively evaluated direct CBCT dose calculations to prove dosimetric accuracy is similar to CT-sim in clinical patients as part of a prospective imaging clinical trial using the C-arm linac equipped with advanced CBCT technology. First, we performed a gamma analysis for volumetric assessment of differences in dose distributions using passing tolerances as a metric. Second, due to known anatomical differences inter- and intra-fractionally and their impact on dose calculation, we performed a correlation analysis of low-HU changes between CT-sim and CBCT with Gamma Pass Rates (GPR) for contextualization of our results. Finally, we performed target and organ-at-risk (OAR) dose-volume comparisons to understand global clinical impact on the treatment plans. To the authors’ knowledge, this study is the first prospective in-vivo advanced CBCT imaging study evaluating dose calculation accuracy on a C-arm linac.

## 2 Materials and methods

### 2.1 Patient imaging and planning

In this study, a C-arm TrueBeam v4.1 linac (Varian Medical Systems, Palo Alto, CA, USA) was equipped with a HyperSight imaging panel (RTI4343iL) for research-only imaging. In preparation for this investigation, scan protocols were calibrated following vendor-supplied guidelines using the Quart phantom to correctly calculate HU values during image acquisition.[15], [16] CBCT images were acquired for a total of 40 patients enrolled with informed consent in an IRB-approved prospective imaging trial (NCT05975619) categorized by general disease site (Supplementary Fig. 1). Of these, 37 (10 head, 7 thorax, 10 abdomen, and 10 pelvis) patients with high-quality imaging were included for the current study. The head treatment site included both intracranial lesions (Brain, 5) and head-and-neck (H&N, 5) diseases. The pelvis protocol was preferred for H&N patients to increase the FOV for inclusion of the shoulders. Three thorax patients were excluded from the current study due to low-dose imaging protocols not suitable for dose calculation or incongruent breath-hold techniques between clinical intent and research imaging. To compensate for the lower number of thorax patients, two patients with two different isocenter treatments were evaluated, resulting in 9 evaluable thorax plans and 39 plans overall. The CT-sim images used in this study were generated from a fan-beam CT (Brilliance Big Bore CT, Philips Healthcare, Andover MS, USA). All treatment plans were computed in RayStation (v12A, RaySearch Laboratories Stockholm, Sweden) on a 2 × 2 × 2 mm^3^ dose grid using the collapsed cone convolution algorithm (v5.7), and subsequently reviewed by a board-certified medical physicist. Planning was performed on TrueBeam, Edge, or Halcyon accelerators (Varian) or the Versa HD (Elekta, Stockholm, Sweden), selected to match the machine used for each patient’s clinical treatment. All plans employed volumetric modulated arc therapy (VMAT) with varying fractionation schemes. The techniques and reconstructions (2 mm slice thickness) used to acquire CBCT images and details about the treatment plans are listed in Supplementary Table 1 per patient case.

### 2.2 Dose calculations and analysis

**Fig. 1:**
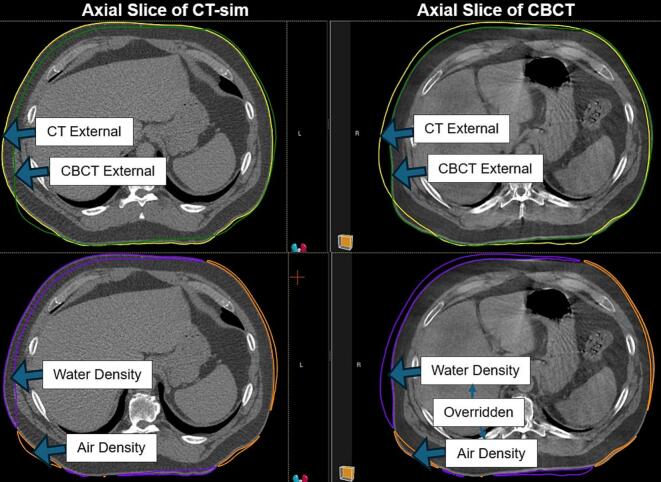
Images from the treatment planning system showing external and material override contours on CT-sim and HSTB-CBCT to account for surface changes.

The research CBCT images were rigidly registered offline with the CT-sim alignment, focusing on the target volume. These registrations were utilized to rigidly transfer target, OAR, and external contours from the CT-sim to CBCT similar to the ARTIA pancreas protocol.[17] To remove the influence of surface and depth variation from tissue changes (e.g., weight loss or positioning differences) on the dose calculation, the CBCT external geometry was modified using Boolean operations to exactly overlap with the patient geometry found in the CT-sim. Without this operation, surface differences would introduce random errors in depth of calculation, possibly confounding the results on inhomogeneity correction accuracy. This was carried out in two steps: first, a region of interest (ROI) was generated by subtracting the CT-sim external contour from the CBCT external contour and assigned a water density override on the CBCT; second, the CBCT external contour was cropped to the intersection of CBCT and CT-sim external contours to exclude any additional tissue volume (Fig. 1). Radiation therapy plans originally calculated on the CT-sim were transferred to the C-arm CBCT using the registration vector and recalculated on the research CBCT without scaling Monitor Units (MU). The CBCT CT number to electron density mappings were assigned based on the tube voltage and current, matching first to voltage and then the closest tube current protocol acquired on the Advanced Electron Density Phantom (Sun Nuclear, Melbourne FL, USA).[15].

For the 3D gamma analysis, the dose calculated on the CT-sim was transferred to the CBCT frame of reference and analysis was performed, with strict 1%/1mm local criteria and low-dose thresholding at 10% and 50% (10%TH, 50%TH), using an in-house python script.[18] The strict criteria were chosen to provide rigorous evaluation highlighting maximal differences in plan calculation. Additionally, a more lenient yet still rigorous 2%/1mm global criteria at 10%TH and 50%TH were used to determine clinical suitability using a passing threshold of 90%, to facilitate comparison with prior studies demonstrating sensitivity to inadequate inhomogeneity corrections[19] and align with the evaluation frameworks outlined in TG-218 and TG-219.[20], [21] To investigate the relationship between CT number matching, anatomical changes, and dose calculation accuracy across scans, the Low Hounsfield Unit Ratio Difference (LHURD) was defined as the absolute difference in percent ratio of voxels with low HU values (<-500HU) relative to all voxels between CBCT and CT-sim. The cut-off value −500HU was primarily chosen for bowel gas,[22] but indicates any excess low intensity areas in CT-sim or CBCT. To restrict the evaluation area, only voxels on the slices within 2 cm superior-inferior to the target and within the CBCT FOV were included for the calculation. The LHURD was then plotted with GPR to perform regression analysis and obtain the coefficient of determination (R^2^) per treatment site.

Since clinical plan quality evaluations often rely on the dose-volume histogram (DVH), several representative metrics were chosen to emulate clinical usage. The highest-dose-level target (GTV, iGTV, or CTV) doses were reported using D99% (near-minimum target dose), D1% (near-maximum hotspot), D_mean_ (average dose), V100%, and V95% (prescription or near-prescription coverage). DVH metrics were also reported for two OARs per case. The OARs chosen for evaluation were a single parallel-like organ evaluated with D_mean_ and a serial-like organ evaluated with D0.03 cc. Parallel-like organs included were: uninvolved brain for the head cases, both parotids in the H&N cases, healthy ipsilateral lung on all slices containing PTV in thorax cases, liver for 9/10 abdomen cases, ipsilateral kidney for one abdomen case far from the liver, and bladder in the pelvis. Serial-like organs included were brainstem in brain cases, spinal cord in H&N, thorax, and abdomen cases, and rectum in pelvis cases.

## 3 Results

**Fig. 2:**
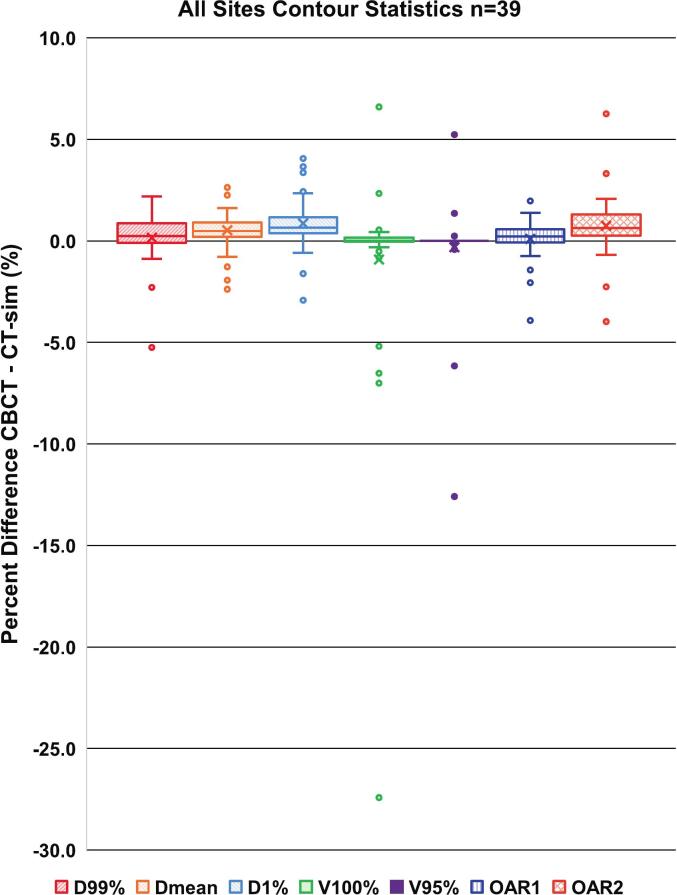
Boxplot with dose-volume statistic percentage differences in CBCT from CT-sim per-case target volume (D99%, D_mean_, D1%, V100%, and V95%), OAR1 (D_mean_), and OAR2 (D0.03 cc).

**Fig. 3:**
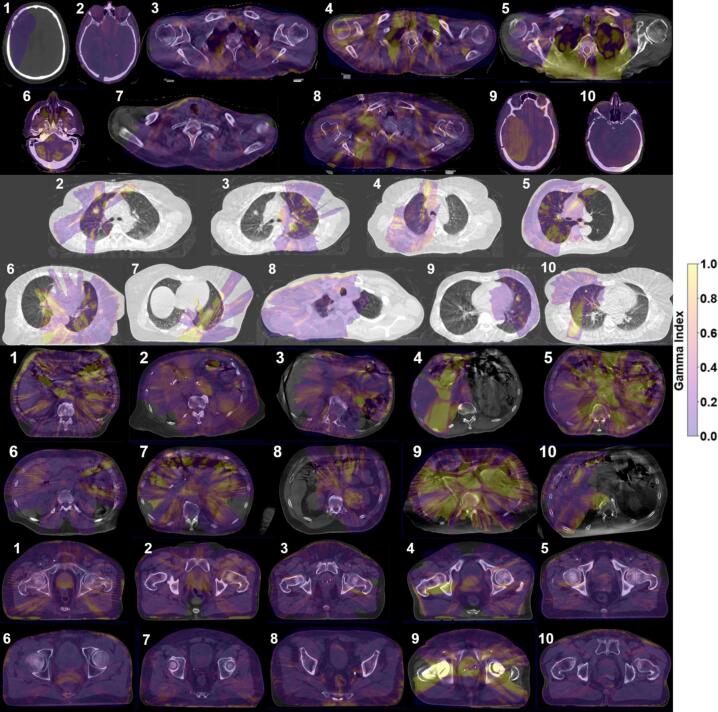
Gamma Index (0–1) overlaid with each case in head (top), thorax (upper-middle), abdomen (lower-middle), and pelvis (bottom) on axial slice with greatest integral dose.

The LHURD, GPR, and contour DVH difference results for the 39 plan calculations are given in Table 1 per body site and DVH differences are summarized in a boxplot (Fig. 2). Gamma value overlays on a single axial CBCT slice for 1%/1mm at 10%TH for each case are given in figure 3 for anatomically informed difference comparisons.

**Table 1:** Statistics for all body site cases (HB=Head, Brain; HN=Head and Neck; T = Thorax; A = Abdomen; P=Pelvis) containing Low Hounsfield Unit (HU) Ratio difference (for pixel values < -500HU), local Gamma Pass Rates (GPR) at 10% thresholding (10TH) and 50% thresholding (50TH), percent difference in dose-volume statistics (D99%, D_mean_, D1%, V100%, and V95%) are given for gross target volume (GTV), internal GTV (iGTV) or clinical target volume (CTV). Percent difference in D_mean_ is given for one organ-at-risk (OAR1) per body site (healthy brain in HB; parotids in HN; nearby healthy lung in T; liver or *ipsilateral kidney in A; bladder in P). Percent difference in D0.03 cc is given for another organ-at-risk (OAR2) per body site (brainstem in HB; spinal cord in HN, T, A; rectum in P).

						**Percent Difference in Dose Statistics (CBCT–CT-sim)**						
**Case**	**Low HU Ratio**	**GPR (%) 2%/1mm**		**GPR (%) 1%/1mm**		**Highest-Dose Level Target Volume**					**OAR 1**	**OAR 2**
		**10TH**	**50TH**	**10TH**	**50TH**	**D99**	**D_mean_**	**D1**	**V100**	**V95**	**D_mean_**	**D0.03**
HB1	0.40	100.0	100.0	100.0	100.0	0.2	0.6	0.6	0.0	0.0	0.8	0.8
HB2	0.04	99.7	100.0	99.6	100.0	0.0	0.0	0.0	0.0	0.0	0.0	0.0
HN3	0.68	99.5	99.5	97.5	98.8	0.1	0.2	0.1	0.0	0.0	0.3	0.3
HN4	0.40	99.1	98.4	91.7	90.8	0.4	0.7	1.4	0.1	0.0	2.0	1.7
HN5	0.70	99.6	99.8	91.1	99.2	−0.3	0.4	0.6	−0.3	−0.1	0.1	0.3
HB6	0.22	100.0	100.0	99.2	98.4	1.3	1.2	1.2	0.0	0.0	1.2	1.1
HN7	0.18	99.7	99.7	99.0	98.7	0.2	0.3	0.5	0.0	0.0	0.1	0.6
HN8	0.24	99.8	99.9	97.2	97.1	1.1	0.9	1.1	0.4	0.0	0.5	−0.3
HB9	0.08	99.7	100.0	99.5	100.0	0.5	0.6	0.6	0.1	0.0	0.5	0.4
HB10	0.03	99.9	100.0	98.2	100.0	0.2	0.1	0.3	0.0	0.0	0.2	−0.2
T2	1.35	99.5	93.4	96.8	92.5	1.4	2.6	4.1	0.0	0.0	1.4	−2.3
T3	2.29	99.7	94.9	96.7	86.9	−2.3	−1.9	−1.6	−27.4	−12.6	−0.7	0.6
T4	0.84	100.0	100.0	98.4	100.0	0.4	0.3	0.4	0.0	0.0	0.1	1.8
T5	1.60	99.8	96.6	95.0	93.4	−2.2	−1.3	−0.6	−5.2	0.0	−0.1	1.9
T6	1.58	99.1	94.2	90.9	83.4	0.2	0.1	0.9	0.0	0.0	−1.4	3.4
T7	2.70	98.9	91.0	90.8	83.5	1.9	2.3	3.7	0.6	0.0	0.7	1.0
T8	3.36	99.6	100.0	98.1	98.7	−0.2	0.4	1.1	0.0	0.0	0.3	1.4
T9	1.07	99.8	97.2	99.4	95.5	−0.7	1.3	2.3	0.0	0.0	−0.1	−0.4
T10	0.19	99.5	100.0	95.1	99.9	0.5	1.6	2.5	0.0	0.0	−0.6	0.3
A1	0.32	99.9	100.0	95.6	96.0	0.9	0.4	0.5	0.2	0.2	0.4	0.4
A2	0.10	99.9	99.2	95.1	96.5	0.9	0.8	0.9	0.2	0.0	0.4	1.6
A3	0.30	100.0	100.0	99.7	100.0	0.5	0.4	0.6	−0.8	−0.4	0.5	0.5
A4	9.31	99.6	95.9	88.4	84.1	−5.2	−2.4	−2.9	−7.0	−6.2	−1.2	−4.0
A5	0.49	99.5	92.1	93.1	71.3	2.2	2.3	2.4	6.6	5.2	−2.1	0.8
A6	0.47	100.0	100.0	99.3	99.7	−0.1	0.6	0.6	0.6	0.3	0.2	1.0
A7	1.99	99.7	99.5	92.5	95.7	−0.1	0.5	0.4	0.5	−0.1	0.6	0.4
A8	0.67	100.0	100.0	96.8	97.6	0.6	0.9	1.0	0.1	0.0	0.5*	1.3
A9	0.74	99.5	88.6	91.5	79.3	0.9	2.4	2.0	2.3	1.4	1.2	2.1
A10	0.51	99.4	97.6	87.7	92.5	0.2	0.4	2.0	−0.5	−0.1	0.6	6.3
P1	0.02	99.8	100.0	99.0	99.5	0.6	0.6	0.8	0.0	0.0	0.7	0.9
P2	0.04	99.7	100.0	98.9	99.9	1.0	0.9	0.9	0.0	0.0	0.6	0.7
P3	0.00	100.0	100.0	99.9	100.0	−0.1	−0.1	0.0	−0.1	0.0	0.2	−0.2
P4	0.08	99.7	100.0	97.2	98.2	0.9	0.8	0.4	−6.5	0.0	−0.3	−0.5
P5	0.19	99.9	100.0	99.2	97.9	0.0	0.5	0.5	−0.2	0.0	−0.3	−0.7
P6	0.82	99.7	99.3	96.6	92.7	0.2	0.3	0.5	0.0	0.0	0.2	0.5
P7	0.01	100.0	100.0	99.3	99.8	0.4	0.6	0.9	0.0	0.0	−0.1	1.1
P8	0.06	99.9	100.0	99.6	99.9	0.8	0.6	0.7	0.3	0.0	−3.9	3.3
P9	0.02	98.5	93.0	89.9	87.5	0.9	1.3	3.4	0.2	0.0	0.5	0.7
P10	0.00	100.0	100.0	99.9	100.0	−0.1	0.0	0.1	0.0	0.0	−0.3	0.3

### 3.1 Gamma Pass rates and low HU Ratio differences

**Fig. 4:**
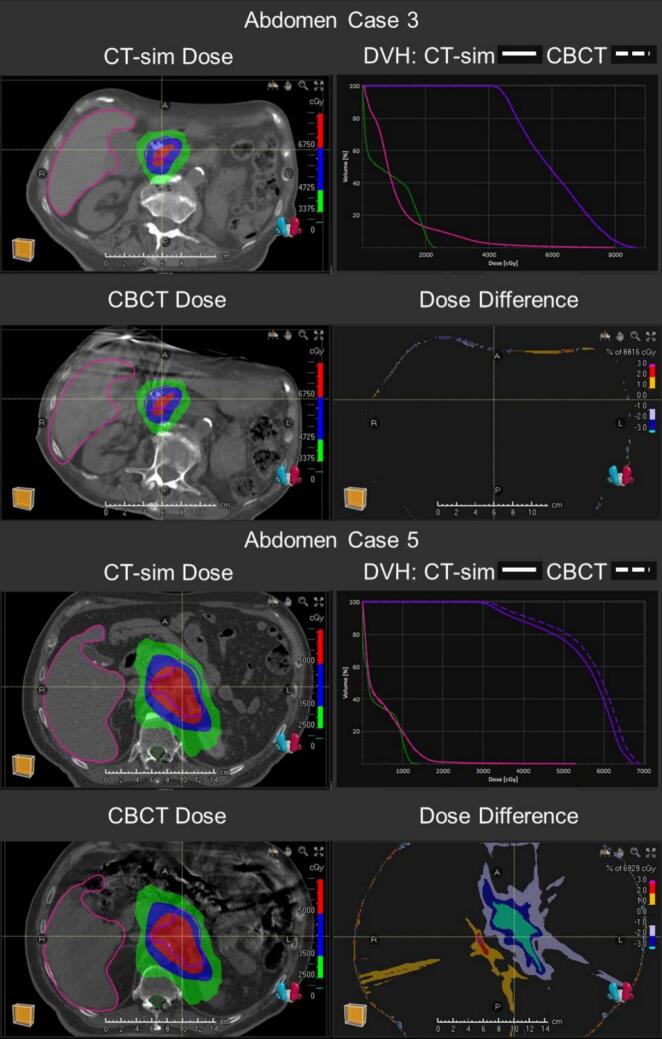
Highest abdominal Gamma Pass Rate (GPR) at 100% (case 3) compared with lowest abdominal GPR at 71.3% (case 5).

Across all body sites, the restrictive 1%/1mm GPR (mean ± standard deviation) were 96.2 ± 3.6% for the 10% TH and 95.0 ± 6.9% for the 50%TH. By treatment site, the 10%TH GPR averaged 97.3 ± 3.2% (head), 95.7 ± 3.1% (thorax), 94.0 ± 4.1% (abdomen), and 97.9 ± 3.0% (pelvis). Under the 50%TH, GPR averaged 98.3 ± 2.8% (head), 92.5 ± 6.6% (thorax), 91.3 ± 9.7% (abdomen), and 97.5 ± 4.2% (pelvis). The lowest GPR was observed in an abdomen plan (case 5) which is contrasted with the highest-performing abdomen case (case 3) in Fig. 4. Using the more lenient 2%/1mm criterion, GPR across all body sites averaged 99.7 ± 0.3% for the 10%TH and 98.2 ± 3.0% for the 50%TH, with only one case (abdomen case 9, 50%TH) falling below the 90% passing threshold.

The LHURD averaged 0.87 ± 1.60% and ranged between 0.00 and 9.31% across all body sites. The regression analysis from plotting 1%/1mm GPR at 10%TH against LHURD yielded R^2^ = 0.202 among all cases and displayed in supplementary Figure 2. When separated by general body site, the R^2^ were 0.372, 0.024, 0.267, and 0.018 for head, thorax, abdomen, and pelvis body sites respectively and given in supplementary Figure 3.

### 3.2 Target and Organ-At-Risk Dose-Volume statistics

**Fig. 5:**
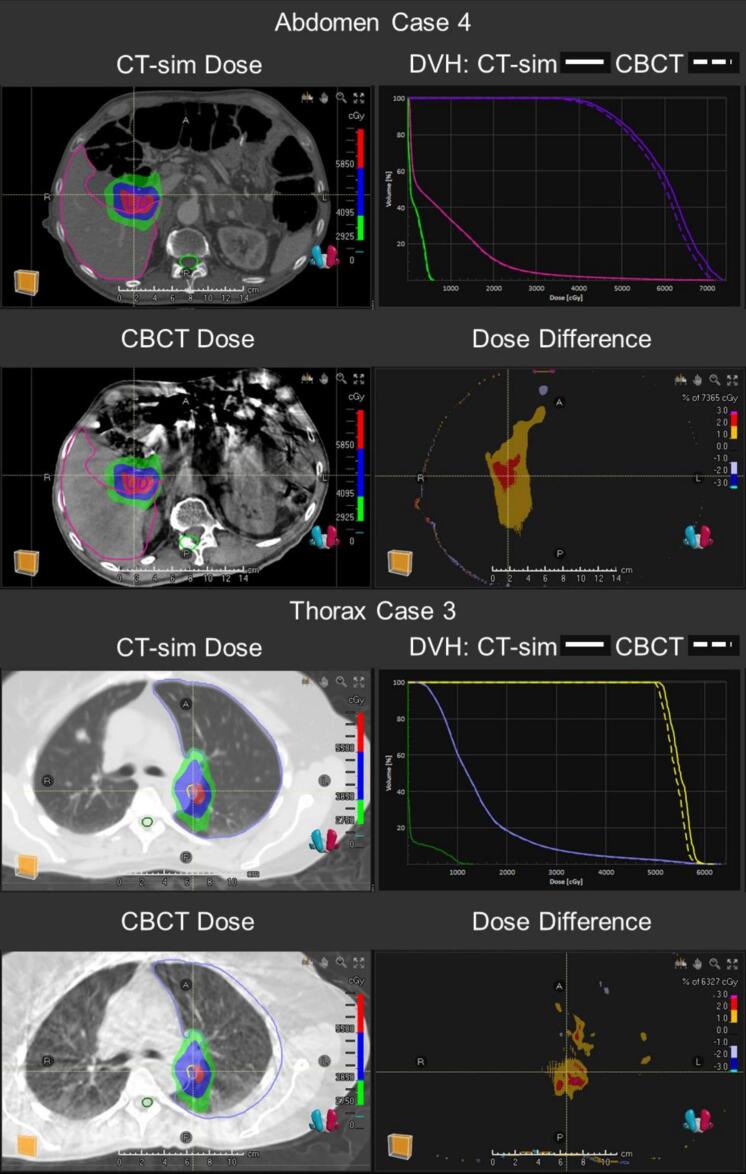
Two worst-case target DVH metric discrepancies caused by the differences in internal anatomy between CT-sim and CBCT in abdomen case 4 (V100%=-7.0%) and thorax case 3 (V100%=-27.4%).

The percentage differences in DVH metrics to the target and OAR volumes are illustrated across all treatment sites in a box plot (Fig. 2). The averaged DVH differences for target volumes were 0.15 ± 1.24%, 0.52 ± 1.01%, 0.86 ± 1.29%, −0.92 ± 4.84%, and −0.31 ± 2.42% for D99%, D_mean_, D1%, V100%, and V95% respectively. Percent differences in OAR DVH metrics averaged 0.10 ± 0.99% and 0.74 ± 1.56% for D_mean_ and D0.03 cc respectively. Abdomen case 4 and thorax case 3, which exhibited the largest percentage differences in DVH metrics, are presented in figure 5 for further visual evaluation.

## 4 Discussion

Direct dose calculation on the advanced C-arm CBCT demonstrated strong overall dose agreement with CT-sim. Even with an exceedingly stringent 1%/1mm local criteria, over 87% of the GPR met the passing criteria above 90% and when using a more clinically reasonable 2%/1mm global criteria, only a single case did not meet the passing criteria. Furthermore, almost all point-dose comparisons were well within the strictest heterogeneous point dose comparison threshold (5%) as set by TG-219.[21] Therefore, the presented results indicate adequate image quality for direct CBCT dose calculation.

GPR were the strongest in the pelvis and head cases where there was more stable and homogenous anatomy compared to abdomen and thorax, which were subject to more inter-fractional changes and heterogeneity. Despite this, the GPR was above 90% for 92% of cases at similar criteria compared to results with prior studies on advanced CBCT, which has received regulatory clearance for direct dose calculation, suggesting that this advanced C-arm CBCT system enables similar quality dose calculations to current clinical direct CBCT dose calculations on O-ring linacs. [11], [19] In the context of the global market, this has the potential to be vastly impactful, leading to more flexible uses of C-arm linacs in a variety of resource settings.[13].

The more lenient 2%/1mm global criteria can be contextualized using secondary dose calculations which use similar evaluation metrics to assess safety within radiotherapy. For example, when using a 3%/3mm global criteria greater than 90%, two separate reports by Kim et al. reported an average GPR of 93.2% when comparing Raystation with Mobius3D (Varian Medical Systems, Palo Alto, CA, USA) and a 5.9% GPR failing rate with MobiusFx.[23], [24] When evaluating with RadCalc (LifeLine Software Inc., Tyler, Tx, USA), Mastella et al., reported stronger GPR with an average of 98.5% with slightly more stringent evaluation criteria of 3%/2mm using a 10% threshold.[25] With our stricter criteria at the 10%TH, 100% of cases passed and all but one of our 50%TH cases passed. The only case below the passing threshold had GPR 88.6%. Direct dose calculation on C-arm CBCT, even with known uncertainties in patient anatomy, is both within the acceptable margins for dose calculation and more precise than commonly used secondary dose calculation tools.

Patient anatomical differences between the CT-sim and CBCT were a driving factor of GPR within this investigation. For example, in thorax plans with lower GPR, motion artifacts caused by involuntary patient motion of the heart and chest wall led to isolated local differences in GPR. Specifically, for lower lung breath-hold patients, subtle differences in diaphragm position affected dose distribution more broadly due to the overall volume of changes in density values in the beam path. In the abdominal cases, dose calculation accuracy may have been affected by contrast injection during CT-sim not present in the CBCT image and incongruence between the bowel gas present in CT-sim and CBCT. The lack of tissue-like material in these regions resulted in radiation field perturbations, negatively impacting overall dosimetry agreement reflected in the GPR. This is directly observed in the lowest GPR case 5 (Fig. 4). One solution to combat this would be to deform the CT-sim anatomy to the CBCT to minimize anatomical differences, which was the mechanism used for dose calculation in previous commercial adaptive platforms.[26] However, there are known reports of the challenges and added uncertainties of deformed CTs for dose calculation that report deviations of up to 15% in target mean dose and 3%/2mm GPR values as low as 36% in controlled phantom studies.[5] Therefore, despite the challenges of comparing dose distribution between CT-sim anatomy with CBCT anatomy, direct CBCT dose calculation may still provide more accurate results in these settings compared to uncontrolled deformed CT-sim images.

Investigating dose calculation differences further, the only pelvis case (9) with GPR under 90% was in a patient with double hip prosthesis. High-density metal implants result in photon starvation and reduced CT number fidelity, impacting dose calculation especially around the edges of the implant. An evaluation on the O-ring unit also identified systematic differences in the HU surrounding metal implants, indicating this discrepancy is not unique to the C-arm CBCT system.[27].

The regression analysis on LHURD and GPR shows weak correlation for head and abdomen body sites, and no correlation for thorax, pelvis, and all sites combined. The greatest LHURD in abdomen case 4 is a direct indication of the large proportion of bowel gas in CT-sim compared to CBCT as observed in Fig. 5. While the primary anatomical changes originally focused investigation around bowel gas and GPR, the correlation is also elevated in the head treatment site. Visual inspection of the highest LHURD in head case 3 and 5 revealed these CBCT scans had greater apparent lung volume near inferior edges of the target. However, these differences appear to be from image artifacts at the edge of the FOV, reducing HU near lung-tissue interfaces rather than true internal anatomy changes. The R^2^ in H&N is therefore elevated because these artifacts detected by LHURD also resulted in lower GPR. Taken together, LHURD is insufficiently sensitive and specific as a surrogate for CBCT dose calculation comparisons with CT-sim. Further investigation and adjustments to thresholds in LHURD or even high-HU ratio differences calculations may help to identify tissue changes specific to each body site, reducing these limitations.

Overall, the DVH metrics in C-arm CBCT dose calculations closely resemble the CT-sim calculations, with 95.4% of the target metrics and 98.7% of the OAR metrics falling within the 5% clinical acceptability criterion. The target in thorax case 3 with the largest deviation in any DVH metric abutted several OARs with steep dose gradients at the edge, causing high sensitivity to positional errors. Here, CBCT motion artifacts also obscured structure edges and inflated CT number, exacerbating registration uncertainties and reducing dose calculation fidelity. Abdomen case 4 contained the next largest DVH metric difference, attributed to excessive bloating at CT-sim abutting the GTV that was not present at CBCT image acquisition (Fig. 5). [5]The only OAR metric outside the acceptability criterion deviated by 6.3% in abdomen case 10 at the spinal cord D0.03 cc. This difference is attributed to the target-focused registration shifting the vertebral bodies, visually indicated by the increased gamma index near the vertebra in Fig. 3. Although several cases gave unfavorable DVH metric differences, in a recalculation setting, these sensitivities true to anatomical changes or registration errors are desired to accurately calculate dose on the CBCT. In deformable CT studies, DVH metrics varied up to 15% in target mean dose in the setting of severe tissue changes, suggesting these results are consistent with prior methods of clinical dose calculation.[5].

The advanced CBCT on a C-arm linac shows promise for in-vivo direct dose calculation with inhomogeneity correction, enhancing the future of patient care. Despite discrepancies caused by tissue changes and positional uncertainty, the stringent criteria gamma analysis and target DVH metrics displayed excellent agreement in comparison to other studies and well within threshold standards for common safety checks within the radiotherapy process. These results demonstrated high-quality C-arm CBCT imaging enables clinicians to make well-informed clinical decisions from direct dose calculations. Future work will investigate the use of advanced CBCT as a primary dataset for contouring, plan optimization, and evaluation.

## CRediT authorship contribution statement

**Kenneth W. Gregg:** Writing – review & editing, Writing – original draft, Visualization, Validation, Supervision, Software, Project administration, Methodology, Investigation, Formal analysis, Data curation, Conceptualization. **Theodore Arsenault:** Writing – review & editing, Visualization, Supervision, Software, Resources, Methodology, Data curation, Conceptualization. **Sagar Regmi:** Writing – review & editing, Investigation, Data curation. **Kyle O’Carroll:** Writing – review & editing, Investigation, Data curation. **Beatriz Guevara:** Writing – review & editing, Investigation, Data curation. **Meiying Xing:** Writing – review & editing, Investigation, Data curation. **Runyon Woods:** Writing – review & editing, Resources, Investigation. **Atefeh Rezaei:** Writing – review & editing, Resources, Project administration, Methodology, Funding acquisition, Conceptualization. **Rojano Kashani:** Writing – review & editing, Resources, Project administration, Methodology, Investigation, Funding acquisition, Conceptualization. **Lauren Henke:** Writing – review & editing, Resources, Project administration, Methodology, Investigation, Funding acquisition, Conceptualization. **Alex Price:** Writing – review & editing, Writing – original draft, Visualization, Validation, Software, Resources, Project administration, Methodology, Investigation, Funding acquisition, Formal analysis, Conceptualization.

## Declaration of competing interest

The authors declare the following financial interests/personal relationships which may be considered as potential competing interests: This research included a prospective imaging clinical trial supported by Varian Medical Systems/Siemens Healthineers. Lauren Henke discloses for Varian: speaker, consulting, institutional grants, and serves on an advisory board; for Elekta: receives honoraria and serves on a clinical advisory board; and receives honoraria from Novartis. The remaining authors do not have any competing interests to disclose.

## Appendix A Supplementary data

Supplementary Data 1 Supplementary Figure S1. Summary of imaging information, plan style, and treatment site per case.

Supplementary Data 2 Supplementary Figure S2. Gamma Pass Rate (GPR) at 1%/1mm and 10% threshold (10%TH) vs. Low Hounsfield Unit (HU) Ratio Difference (LHURD) for all body sites with displayed coefficient of determination (R 2 = 0.202).

Supplementary Data 3 Supplementary Figure S3. Gamma Pass Rate (GPR) at 1%/1mm and 10% threshold (10%TH) vs. Low Hounsfield Unit (HU) Ratio Difference (LHURD) separated by general body site with coefficient of determination (R 2 ) displayed on each panel.
