# Supplementary material for: First evaluation of in-patient dose calculation accuracy on a C-arm Linear Accelerator with advanced Cone-Beam computed tomography (CBCT) imaging
Source: Phys Imaging Radiat Oncol. 2026 May 13;39:100991. doi: 10.1016/j.phro.2026.100991 (PMC13200127; doi:10.1016/j.phro.2026.100991)
Supplement: Supplementary Data 1 — Supplementary Figure S1. Summary of imaging information, plan style, and treatment site per case. [file mmc1.docx]

| Body Site | Case | Protocol (kV, mAs) | Reconstruction | Plan Style | Treatment Site |
| --- | --- | --- | --- | --- | --- |
| Head | 1 | Head P (125, 620) | iCBCT-MAR | 2-target SRS | Brain |
|  | 2 | Head P (125, 370) | iCBCT-MAR | VMAT | Brain |
|  | 3 | Pelvis (125, 1060) | iCBCT-MAR | 3-level VMAT | H&N |
|  | 4 | Pelvis (125, 1060) | iCBCT-MAR | 3-level VMAT | H&N |
|  | 5 | Pelvis (125, 1060) | iCBCT-MAR | 3-level VMAT | H&N |
|  | 6 | Head P (125, 410) | iCBCT-MAR | 5-target FSRT | Brain |
|  | 7 | Pelvis (125, 1060) | iCBCT-MAR | 3-level VMAT | H&N |
|  | 8 | Pelvis (125, 810) | iCBCT-MAR | 2-level VMAT | H&N |
|  | 9 | Head P (125, 310) | iCBCT-MAR | VMAT | Brain |
|  | 10 | Head P (125, 410) | iCBCT-MAR | VMAT | Brain |
| Thorax | 1 | PelvisLg (140, 1330) | iCBCT-MAR | VMAT, BH | R lower lung |
|  | 2 | Thorax (125, 670) | iCBCT-MAR | SBRT, FB | R upper lung |
|  | 3 | Thorax (125, 670) | iCBCT-MAR | SBRT, FB | L upper lung |
|  | 4 | Thorax (125, 670) | iCBCT-MAR | SBRT, FB | R upper lung |
|  | 5 | PelvisLg (140, 1670) | iCBCT-MAR | VMAT, BH | R upper lung |
|  | 6 | Thorax (125, 670) | iCBCT-MAR | VMAT, FB | L lung |
|  | 7 | Pelvis (125, 1080) | iCBCT-MAR | SBRT, BH | L lower lung |
|  | 8 | Pelvis (125, 1060) | iCBCT | SBRT, FB | R upper lung |
|  | 9 | Pelvis (125, 1060) | iCBCT | SBRT, FB | L lung |
|  | 10 | Pelvis (125, 1240) | iCBCT-MAR | SBRT, FB | R middle lung |
| Abdomen | 1 | Pelvis (125, 2010) | iCBCT-MAR | SBRT | Pancreas |
|  | 2 | PelvisLg (140, 1700) | iCBCT-MAR | SBRT | Nodes |
|  | 3 | PelvisLg (140, 1700) | iCBCT-MAR | VMAT | Pancreas |
|  | 4 | PelvisLg (140, 1700) | iCBCT-MAR | VMAT | Nodes |
|  | 5 | PelvisLg (140, 1710) | iCBCT-MAR | SBRT | Pancreas |
|  | 6 | PelvisLg (140, 1730) | iCBCT-MAR | SBRT | Pancreas |
|  | 7 | PelvisLg (140, 1710) | iCBCT-MAR | SBRT | Pancreas |
|  | 8 | PelvisLg (140, 1700) | iCBCT-MAR | SBRT | Kidney |
|  | 9 | PelvisLg (140, 1710) | iCBCT | SBRT | Pancreas |
|  | 10 | PelvisLg (140, 1700) | iCBCT | VMAT | Liver |
| Pelvis | 1 | PelvisLg (140, 1500) | iCBCT-MAR | SBRT | Prostate |
|  | 2 | Pelvis (125, 1330) | iCBCT-MAR | VMAT | Prostate |
|  | 3 | PelvisLg (140, 1680) | iCBCT-MAR | SBRT | Prostate |
|  | 4 | PelvisLg (140, 1680) | iCBCT-MAR | VMAT | Prostate |
|  | 5 | PelvisLg (140, 1680) | iCBCT | SBRT | Prostate |
|  | 6 | PelvisLg (140, 1680) | iCBCT-MAR | VMAT | Prostate+Nodes |
|  | 7 | PelvisLg (140, 1680) | iCBCT-MAR | VMAT | Prostate+Nodes |
|  | 8 | PelvisLg (140, 1680) | iCBCT | VMAT | Prostate+Nodes |
|  | 9 | PelvisLg (140, 1680) | iCBCT-MAR | SBRT | Prostate |
|  | 10 | PelvisLg (140, 1680) | iCBCT-MAR | SBRT | Prostate |
