# Supplementary figures and images for: First evaluation of in-patient dose calculation accuracy on a C-arm Linear Accelerator with advanced Cone-Beam computed tomography (CBCT) imaging
Source: Phys Imaging Radiat Oncol. 2026 May 13;39:100991. doi: 10.1016/j.phro.2026.100991 (PMC13200127; doi:10.1016/j.phro.2026.100991)

## 1%/1mm Gamma Pass Rate at 10% threshold vs. Low HU Ratio Absolute Difference

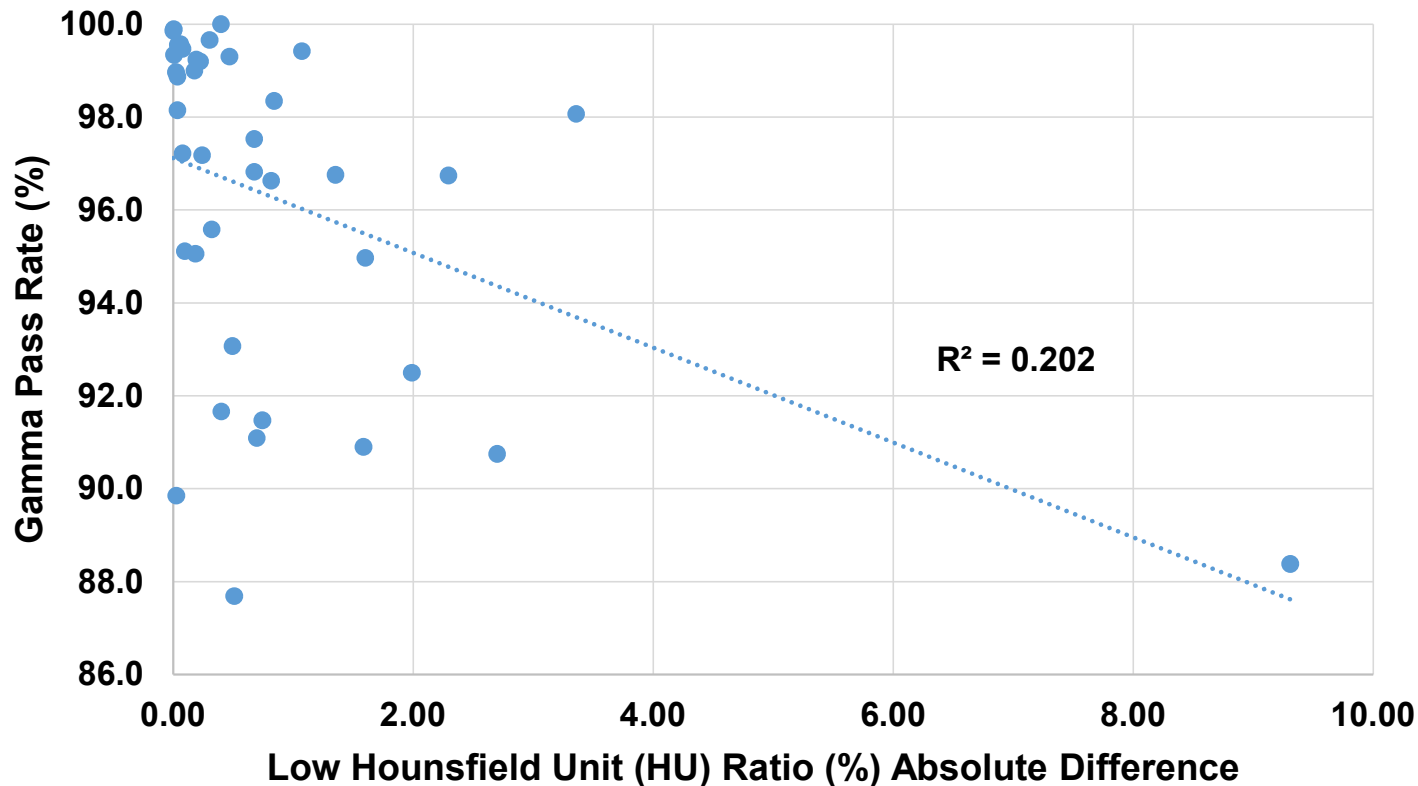

Supplement: Supplementary Data 2 — Supplementary Figure S2. Gamma Pass Rate (GPR) at 1%/1mm and 10% threshold (10%TH) vs. Low Hounsfield Unit (HU) Ratio Difference (LHURD) for all body sites with displayed coefficient of determination (R 2 = 0.202). [file mmc2.pdf]
