# Supplementary material for: First evaluation of in-patient dose calculation accuracy on a C-arm Linear Accelerator with advanced Cone-Beam computed tomography (CBCT) imaging
Source: Phys Imaging Radiat Oncol. 2026 May 13;39:100991. doi: 10.1016/j.phro.2026.100991 (PMC13200127; doi:10.1016/j.phro.2026.100991)
Supplement: Supplementary Data 3 — Supplementary Figure S3. Gamma Pass Rate (GPR) at 1%/1mm and 10% threshold (10%TH) vs. Low Hounsfield Unit (HU) Ratio Difference (LHURD) separated by general body site with coefficient of determination (R 2 ) displayed on each panel. [file mmc3.pdf]

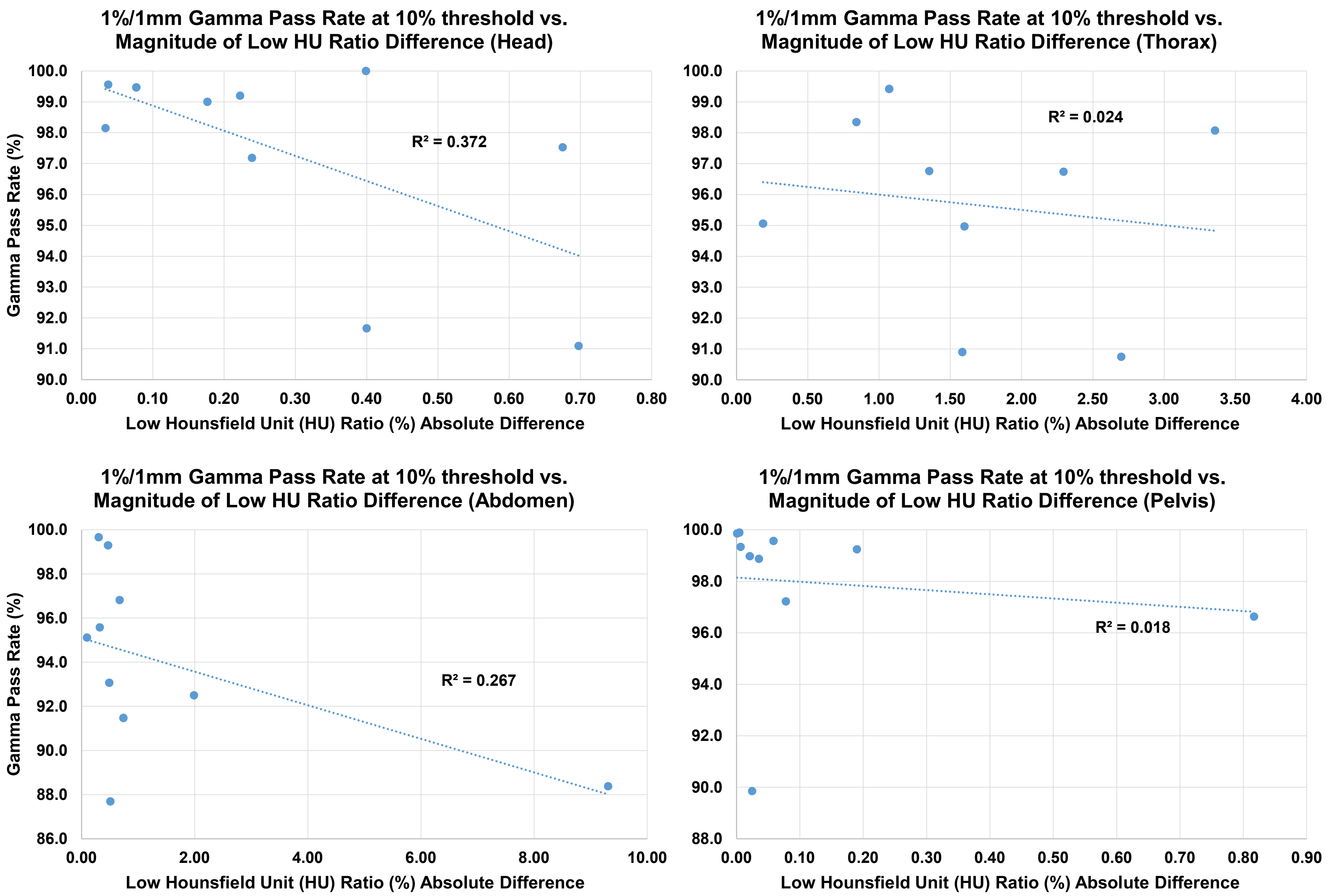
1%/1mm Gamma Pass Rate at 10% threshold vs. Magnitude of Low HU Ratio Difference (Head)
Gamma Pass Rate (%)
Low Hounsfield Unit (HU) Ratio (%) Absolute Difference
R² = 0.372
1%/1mm Gamma Pass Rate at 10% threshold vs. Magnitude of Low HU Ratio Difference (Thorax)
Low Hounsfield Unit (HU) Ratio (%) Absolute Difference
R² = 0.024
1%/1mm Gamma Pass Rate at 10% threshold vs. Magnitude of Low HU Ratio Difference (Abdomen)
Gamma Pass Rate (%)
Low Hounsfield Unit (HU) Ratio (%) Absolute Difference
R² = 0.267
1%/1mm Gamma Pass Rate at 10% threshold vs. Magnitude of Low HU Ratio Difference (Pelvis)
Low Hounsfield Unit (HU) Ratio (%) Absolute Difference
R² = 0.018
